# Guidewire-Assisted Reduction Technology Combined with Postural Reduction Improves the Success Rate of Internal Vein Catheterisation

**DOI:** 10.1155/2022/7171578

**Published:** 2022-05-17

**Authors:** Feng-Xian Li, Yan-Ping Li, Hong-Yang Zhang, Mei-Jing Tian, Huan-Huan Gao, Gui-Juan Zhang, Peng Su

**Affiliations:** Department of Oncology Beijing Shijingshan Hospital, Capital Medical University, Beijing 100043, China

## Abstract

**Objective:**

To investigate the value of guidewire-assisted reduction technology (which increases the stiffness of a catheter through the use of a guidewire, thereby protecting the puncture point and distal vein from breakage) combined with postural reduction for malpositioned catheters in the internal jugular vein during peripherally inserted central venous catheter catheterisation.

**Methods:**

From January 2015 to August 2020, we used ultrasound to perform guided puncture and monitoring. We identified the tip of the catheter as malpositioned in the internal jugular vein in 99 patients during the catheterisation process. These patients were divided randomly into a control group and an experimental group. In the control group, 43 cases received guidewire-assisted reduction technology, while in the experimental group, 56 patients received guidewire-assisted reduction technology combined with an upright posture. This study compared the efficacy of these two methods.

**Results:**

The results showed that 30 catheters were reduced successfully in the control group, with a success rate of 69.8%. In the experimental group, 53 cases were successfully reduced, with a success rate of 94.6%. The catheter reduction success rate in the experimental group was significantly higher than in the control group; this was a statistically significant difference (*P*=0.001).

**Conclusion:**

Guidewire-assisted reduction technology combined with postural reduction can improve the success rate of the reduction of malpositioned catheters in the internal jugular vein.

## 1. Introduction

A peripherally inserted central venous catheter (PICC) provides patients with a high-quality intravenous infusion channel, which is widely used in chemotherapy or nutrient solution infusion processes for cancer patients. The lumen is often open, which provides favourable anatomical conditions for the malposition of the catheter. Since no two patients' blood vessel anatomies are identical, anatomical variations will exist, which means the angle at which the subclavian vein merges into the brachiocephalic vein will differ.

A malpositioned PICC is a very common problem, with an incidence rate of 1.2%–10%. The malpositioning of the catheter tip in the jugular vein is the most important of such problems [1–7]. The incidence of the malposition of the catheter tip in the jugular vein accounts for 30.3% of the total number of malpositioned cases [1]. When the catheter tip is in the wrong position, it may lead to bleeding and infection at the puncture site as well as other complications, such as catheter prolapse or rupture.

Once a catheter has been identified as malpositioned, it is necessary to perform a catheter redirection operation to ensure the catheter tip position normalises, thereby reducing the occurrence of catheter-related complications. At present, there are many methods for malpositioned PICC reduction, with the reduction success rate varying from 64% to 100% [2, 4, 8, 9]. There are many ways to adjust a malpositioned PICC in the internal jugular vein [2, 4, 8, 10–12], including the guidewire-assisted reduction technique, which has a reported success rate of 64%–93% [2, 4, 13, 14], and the position reduction method, with a success rate of 86% [2, 8, 9]. The 2016 version of the US infusion therapy guidelines states that in the case of a malpositioned catheter, a guidewire-assisted reduction can be performed within the intervention department, and fluoroscopy can be used to reposition the catheter [15]. Posture also affects PICC [16–18], so postural reduction for malpositioned catheters can also be used in PICC catheterisation.

Therefore, we conducted this study to investigate the value of guidewire-assisted reduction technology combined with postural reduction for treating malpositioned catheters in the internal jugular vein during PICC catheterisation.

## 2. Materials and Methods

### 2.1. Subjects

From January 2015 to August 2020, patients whose catheter tip was malpositioned in the internal jugular vein were recruited and divided randomly into a control group (guidewire-assisted reduction technique) and an experimental group (guidewire-assisted reduction technique combined with postural reduction). The patients received random cards according to the order of their visit and were assigned to the control group or experimental group according to the numbers on the random cards. The efficacy of these two methods was compared in this study, which was conducted in accordance with the Declaration of Helsinki and approved by the Ethics Committee of the Beijing Shijingshan Hospital (2020-08). All participants provided signed informed consent.

### 2.2. Inclusion and Exclusion Criteria

The inclusion criteria were informed consent for PICC catheterisation had been obtained, the catheter was malpositioned in the internal jugular vein during PICC catheterisation, the patient was capable of postural adjustment, the patient was older than 18 years, and the patient had provided signed informed consent.

The exclusion criteria were patients with malpositioned catheterisation in blood vessels other than the internal jugular vein, patients with catheter infusion failure, patients who were unable to adjust their position and patients with mental disorders, and patients whose data were incomplete.

### 2.3. Peripherally Inserted Central Venous Catheter Catheterisation Method

This study used a 4Fr catheter (type of catheter: 7655405), which was obtained from Bard Access Systems Inc., 605 North 5600 West Salt Lake City, Utah, 84116, USA. The operation process was carried out in accordance with the PICC operation specifications in the infusion therapy guide [15]. The PICC catheter was inserted using ultrasonic guidance and Seldinger manipulation. Various procedures were conducted in the following sequence: ex vitro measurement of catheter insertion length and arm circumference, routine disinfection and placement of sterile bed sheet, puncture under ultrasonic guidance and insertion of guidewire after returning blood, expansion of the skin with a sharp knife after local anaesthesia, insertion of vascular sheath, delivery of catheter to premeasured length, and electrocardiogram (ECG) positioning to check the catheter tip position. The veins were accessed using a bedside ultrasound device (E-CUBE 5, Alpinion Medical Systems Co., Ltd., South Korea), and the catheter was placed using a modified Seldinger technique.

If abnormal conditions were identified, an ultrasound examination of the subclavian vein and internal jugular vein was performed to confirm the presence or absence of a catheter; if the catheter was malpositioned, it was adjusted under ultrasonic monitoring.

### 2.4. B-Ultrasound Examination Assistance

B-ultrasound was used to determine the malpositioned situation of the internal jugular vein catheter during the PICC catheterisation process, to monitor the withdrawal of the internal jugular vein catheter, and to confirm whether a catheter was present in the internal jugular vein following the reduction operation.

### 2.5. Catheter Reduction Method

In the control group (guidewire-assisted reduction technology), the tip of the catheter was withdrawn from the internal jugular vein under ultrasonic monitoring; the operator withdrew the guidewire by 3–5 cm and slowly pushed the catheter (including the guidewire) to the estimated length prior to an ultrasound examination of the internal jugular vein. This operation could be repeated two to three times until it was confirmed by ultrasound examination that there was no catheter in the internal jugular vein. In the experimental group (guidewire-assisted reduction combined with postural reduction technology), the catheter tip was withdrawn from the internal jugular vein under B-ultrasound monitoring; an assistant helped the patient to raise the head of the bed by 90° to a high Fowler position [8] before the surgeon withdrew the guidewire by 3–5 cm, slowly pushing the catheter (including the guidewire) to the estimated length. Ultrasound checks were performed on the internal jugular vein. This operation could be repeated two to three times until the ultrasound examination confirmed that there was no catheter in the internal jugular vein.

### 2.6. Efficacy Assessments

#### 2.6.1. Successful Catheter Adjustment

The ultrasound monitoring confirmed that the catheter was not in the internal jugular vein, and a postoperative chest X-ray confirmed that the tip of the catheter was not in the internal jugular vein but the superior vena cava.

#### 2.6.2. Failed Catheter Adjustment

The ultrasound monitoring indicated that the catheter was malpositioned in the internal jugular vein during the catheterisation process; thus, the catheter was adjusted two to three times using the corresponding method. Ultrasound monitoring or postoperative X-rays confirmed that the catheter remained in the internal jugular vein.

### 2.7. Statistical Analysis

We used SPSS 19.0 (IBM, Chicago, USA) software to conduct the statistical analysis. The continuous variables of normal distribution were expressed as mean ± standard deviation, the continuous variables of nonnormal distribution were expressed as median (interquartile range), and the categorical variables were expressed as frequency (percentage (%)). For two comparisons, each value was compared by a *t*-test when each datum conformed to a normal distribution, while the nonnormally distributed continuous data were compared using nonparametric tests. The counting data were tested by the chi-square test. A value of *P* < 0.05 was considered statistically significant.

## 3. Results

### 3.1. General Characteristics

From January 2015 to August 2020, there were 1162 cases involving catheterisation; among these were 11 cases of failure (success rate of 99.05%). The ultrasound monitoring conducted during catheterisation indicated that the tip of the catheter was malpositioned in the internal jugular vein in 99 cases (incidence rate of 8.60%).

The control group had 43 cases, including 22 males and 21 females (age range: 48–71 years). There were 28 cases of lung cancer, 8 cases of bowel cancer, 4 cases of oesophageal cancer, and 3 cases of liver cancer. In terms of blood vessel puncture, there were 18 cases of right basilic vein, 12 cases of left basilic vein, 8 cases of right brachial vein, and 5 cases of left brachial vein.

The experimental group had 56 cases, including 32 males and 24 females (age range: 33–91 years). There were 19 cases of lung cancer, 15 cases of bowel tumours, 12 cases of breast cancer, and 10 cases of ovarian cancer. In terms of blood vessel puncture, there were 19 cases of right basilic vein, 16 cases of left basilic vein, 15 cases of right brachial vein, and 6 cases of left brachial vein.

### 3.2. The Main Outcomes

The results showed that 30 catheters were reduced successfully in the control group, with a success rate of 69.8%. In the experimental group, 53 cases were reduced successfully, with a success rate of 94.6%. The chi-square test outcomes were 11.10. The *P* value of the four-grid test result was 0.001, which was statistically significant. The catheter reduction success rate in the experimental group was significantly higher than in the control group, as given in Table 1.

## 4. Discussion

The 2016 version of the US infusion therapy guidelines states that in the case of a malpositioned catheter, a guidewire-assisted reduction can be performed within the intervention department, and fluoroscopy can be used to reposition the catheter [15]. The use of a guidewire can improve the hardness and toughness of the catheter. However, the direction of the advancement of the catheter tip in the internal jugular vein remains unpredictable. In vessels close to the central vein, changes in haemodynamics have a major impact on catheter placement. A study by Cohen et al. [16] showed that changes in the intravascular pressure gradient are significantly different in different postures, such as sitting and standing, and the greater the height difference, the greater the pressure gradient, and thus, the greater the local hydrostatic pressure. Kubota et al. [18] compared haemodynamic changes in different Fowler positions and found that the cardiac stroke volume and preload were higher in the upper torso 60° upright position than in the full torso 60° upright position. This means that there is greater central venous inflow and faster flow in the Fowler position than in the supine position. These studies provide a haemodynamic rationale for the catheter reduction approach used in this study.

A study by Spencer [8] highlights a method for improving catheter repositioning using high-flow irrigation techniques. The high-flow flush technique can be described as a rapid manual flush using 10–20 cc of 0.9% sterile sodium chloride administered aseptically through a catheter via the distal lumen. It is worth noting that in this technique, the upper half of the patient's body is between 60° and 90° relative to the lower half of their body; the patient is encouraged to perform several deep coughs prior to the administration of the flush, which is shown in the corresponding video. Natural changes in intrathoracic pressure allow for catheter movement within the vessel due to changes in both vessel size and flow dynamics. This process is repeated for every flush attempt. A total of 86% (46/53) of catheters were successfully repositioned on the first high-flow flush attempt. The method used in this study has a higher reduction success rate compared with previously described methods of catheter reduction.

The position of the body was considered in our research, which is consistent with Spencer's study. The high-flow flush technique facilitates the reduction of the catheter tip by rapidly injecting fluid, causing the catheter tip to swing. In this study, the proximal portion of the catheter was made flexible due to the guidewire, thus avoiding catheter buckling or knotting. By retracting the guidewire, since the part near the tip is soft, it is easy to reposition in accordance with the direction of blood flow. Compared with the method described by Spencer, our method is more active and controllable. The retracted length of the guidewire can be adjusted actively at any time as required, so the success rate of repositioning is higher.

This method differs from The 2016 Infusion Therapy Standards of Practice [15], in which the recommended catheter reduction methods include head elevation and ambulation. In the present study, the Fowler position was used instead of raising the head. The Fowler position can significantly increase the central venous pressure difference, and haemodynamic changes are more obvious. In addition, the recommended catheter reduction method in The 2016 Infusion Therapy Standards of Practice includes invasive techniques, i.e., the catheter is withdrawn, advanced, and withdrawn during catheterisation. The control group in this study were treated using the invasive technique recommended by the guidelines. The results demonstrate that the Fowler position combined with the guidewire technique can achieve a higher rate of catheter reduction.

The innovation of this research method lies in ascertaining the malpositioned location of the catheter through the application of ultrasound examination during the operation, performing the adjustment operation in real time, and immediately evaluating its effects. This method saves time and resources and can be used for positioning and monitoring. The most critical period for avoiding and correcting a malpositioned catheter is during the catheterisation process. Intraoperative ultrasound can rapidly detect a malpositioned catheter in the internal jugular vein, and it can be used to monitor the adjustment operation. Finally, the effectiveness of adjusting the catheter can be evaluated. Therefore, intraoperative ultrasound assistance is of great significance to the malpositioned catheter reduction operation and is a comparatively simple, convenient, accurate, and effective method [3, 19, 20].

The catheter reduction method reported in this study uses a combination of the guidewire-assisted reduction technique and postural reduction. Guidewire-assisted reduction technology increases the stiffness of a catheter through the use of a guidewire. The tip of the catheter supported by the guidewire in withdrawal is very soft and floats easily to the lower segment of the superior vena cava (ideal position) in accordance with the direction of the blood flow. In this study, the high Fowler position reduction method significantly increased stroke volume, increased return blood volume, and accelerated blood flow via the changes in body position, thus promoting the malpositioned catheter in the internal jugular vein to flow back to the superior vena cava. The success rate of the catheter adjustment was significantly higher than when using the guidewire-assisted reduction technique alone. This was especially the case for the 13 patients from the control group in which the malpositioned internal jugular vein catheter reduction failed. The combination of the guidewire-assisted reduction technique and postural reduction not only prevented the catheter from being broken in the vein where the puncture point was located but also ensured the softness of the catheter tip. Overall, with the help of rapid venous return to the catheter, the catheter was reduced to the ideal position.

This method failed in three cases. In one case, the malpositioned catheter was not identified via ultrasound examination following catheter reduction, while the postoperative X-ray confirmed that the catheter remained in the internal jugular vein. In the second case, the catheter was not identified in the ipsilateral internal jugular vein by ultrasound monitoring after catheter reduction, while the postoperative X-ray confirmed that the catheter was in the contralateral internal jugular vein. In the last case, the patient was unable to tolerate the end sitting position, which resulted in an incorrect body position. Therefore, the method used in this study requires proficiency in B-ultrasound operation and diagnostic techniques. During the operation, B-ultrasound monitoring is required to check the internal jugular vein on both sides; additionally, an upright sitting position must be maintained for a period of time.

There were several limitations in this study. First, this research was a single-centre trial, so a multicentre trial will be needed in the future. Second, the sample size of this study was limited, so a larger trial with more participants will be necessary.

This research is a preliminary study. During PICC catheterisation, the translocation of the catheter in the internal jugular vein was detected in time by ultrasound and ECG technologies. When an ectopic catheter is located, guidewire-assisted reduction technology combined with postural reduction is used to reset the catheter in time, and ultrasound and ECG technologies are used to confirm whether the catheter is in a suitable position. The combined application of various techniques solves the problem of PICC catheter translocation in the internal jugular vein during catheterisation rather than postoperatively identifying catheter translocation by X-ray.

## Figures and Tables

**Table 1:** Comparison of two reset methods.

Group	Success	Fail	*X* ^2^	*P*

Control group	30	13	11.10	0.001
Observation group	53	3		

## Data Availability

The datasets used and analyzed during the current study are available from the corresponding author upon request.
